# Psychological responses and dietary changes of residents during the local outbreak of COVID-19 in the post-epidemic era: A cross-sectional study

**DOI:** 10.1097/MD.0000000000032792

**Published:** 2023-02-03

**Authors:** Luying Qi, Qingtao Yu, Zhengyan Liang, Yang Lu, Zhihua Ma, Chujie Hou, Zhiyong Zhu, Liyong Chen

**Affiliations:** a Department of Emergency Management, School of Public Health, Cheeloo College of Medcine, Shandong University, Jinan, China; b Department of endocrinology, The People’s Hospital of Huaiyin Jinan, Jinan, China; c Department of Nutrition, The Second People’s Hospital of Shandong Province, Jinan, China; d Department of Nutrition and Food Hygiene, School of Public Health, Shandong First Medical University, Jinan, China; e Department of Surgery, Shandong Rehabilitation Hospital, Jinan, China; f Department of Nutrition, Qilu Hospital, Shandong University, Jinan, China.

**Keywords:** anxiety, COVID-19, depression, dietary changes, fear of COVID-19, health status, lifestyle changes, local outbreak, lockdown or quarantine, post-epidemic era

## Abstract

The coronavirus disease 2019 (COVID-19) pandemic has had a dramatic impact on the psychological state and dietary behavior of individuals. Many previous studies have discussed the psychological and dietary problems during the first COVID-19 pandemic. However, few papers have discussed them during the local COVID-19 outbreak in the post-epidemic era. To explore the psychological responses and the influencing factors, dietary changes and the relationship with psychological responses during the local COVID-19 outbreak in the post-epidemic era. Methods: A total 3790 residents were surveyed by online questionnaire to collect information about social demography, health status, local outbreak related information, lifestyle changes, anxiety and depression. Binary logistic regression was used to discuss the influencing factors of anxiety and depression. Kendall tau-b correlation coefficient was used to discuss the relationship between anxiety, depression and dietary changes. Self-perceived physical condition, chronic disease, lockdown or quarantine, fear of COVID-19, changes in smoking, drinking and physical activity were the influencing factors of anxiety and depression. The top 3 foods with increased intake were drinking water, fresh fruits and fresh vegetables, while the top 3 foods with reduced intake were puffed foods, fried foods and sugary foods. Dietary changes were correlated with generalized anxiety disorder-7 and patient health questionnaire-9 scores. These findings provide experience and clues for local governments to improve the psychological status and dietary habits of residents during the local COVID-19 outbreak in the post-pandemic era.

## 1. Introduction

The coronavirus disease 2019 (COVID-19) is an acute highly contagious respiratory syndrome. It was first diagnosed in Wuhan, China in late 2019. Since then, COVID-19 has rapidly spread around the world and seriously threatened human health and life.^[1,2]^ On March 11, 2020, the World Health Organization (WHO) declared COVID-19 a global pandemic due to the rapidly increasing number of newly reported confirmed cases and deaths.^[3]^ In order to protect people lives, countries around the world have adopted a series of protective/distancing measures such as quarantine, social isolation, lockdown, and movement restrictions to prevent the spread of the virus.^[1,4,5]^

In China, 3 weeks after the outbreak of the Wuhan epidemic, the Chinese government responded quickly to stop the spread of the virus and imposed a lockdown on Wuhan on January 23, 2020.^[6]^ Home quarantine of uninfected ordinary people was considered the best way to eliminate the chance of infection.^[7]^ Many countries took measures to close enterprises, public places, restaurants and parks, suspend flights and close airports and borders, aiming to ensure social distance, limit population mobility, and mitigate the impact of the pandemic.^[8]^ However, the viral spreading, the social distancing and the enforced isolation in the early stage of COVID-19 have all adversely affected the mental health of the population.^[8–10]^ In the initial phase of COVID-19 outbreak in China, more than half of the respondents rated the psychological impact as moderate to severe, and about 1-third reported experiencing moderate to severe anxiety disorders.^[6]^ During the COVID-19 pandemic, the prevalence of depressive symptoms in the United States increased more than therefold, from 8.5% before COVID-19 to 27.8% during COVID-19.^[11]^ In a survey of 204 countries, an increase in the prevalence of severe depression and anxiety during the early phase of the pandemic was associated with an increase in SARS-CoV-2 infection rates and a decrease in population mobility.^[12]^ Longitudinal studies of British adults have observed a more than 50% increase in mental health problems during the pandemic compared to before.^[13]^ The percentage of people with high and very high levels of psychological distress increased in Italy in the early stage of the COVID-19 pandemic.^[14]^ Many studies have also found that during the COVID-19 pandemic, fear of COVID-19, history of chronic diseases, smoking, drinking, and physical activity were all influencing factors of psychological problems of the public.^[14–16]^

Measures such as quarantine, restrictions on population mobility, staying at home, lockdown, state of emergency, entry restrictions to the country, and suspension of international flights led to changes in people dietary behavior and food intake, which were greatly affected by COVID-19.^[17,18]^ In the early stage of the COVID-19 outbreak in China in 2019, an increase in residential intake was most commonly reported for vegetables, water and fruits, while a decrease was most commonly reported for sea cucumber, fish, shrimp and crab, and poultry meat.^[19]^ During the outbreak of COVID-19, the nutritional style of Iranians turned to healthier foods.^[20]^ Participants in Türkiye reported an increased consumption of fresh fruits and vegetables, eggs, milk, and red meat. The consumption of junk foods (such as biscuits, french fries, and chocolates) and carbohydrate (such as cakes, desserts, and bread) decreased.^[21]^ A study on the dietary habits of Spain during the lockdown summarized that compared with the habits before the lockdown, residents had healthier foods choices. Many residents reduced their intake of fried foods, snacks, fast foods, red meat, cakes and sweet drinks, and increased their intake of olive oil, vegetables, fruits or beans.^[22]^ During the first lockdown in Germany, the intake of fruits, vegetables, nuts, fish and eggs, and unhealthy foods (such as processed foods) by residents increased more significantly than that of decreased.^[23]^ In Poland, a large percentage of people have changed their eating habits and started eating and snacking more.^[24]^ A study in Italy showed that during the period of isolation, the intake of packed red meat, processed meat, biscuits and smeared cream increased, the intake of fruits and vegetables insufficient, and the consumption of fresh milk decreased.^[25]^ Sidor A found that frequent exposure to unconfirmed statements and news about COVID-19 might aggravate the impact of forced isolation on anxiety and fear. These psychological barriers might lead to overeating, especially the intake of high calorie, and high sugar foods.^[14]^ Ben Hassen^[26]^ pointed out that anxiety, boredom and psychological reactions to isolation might lead to dramatic changes in lifestyle, including overeating, and not caring about food quality. Therefore, the quarantine and lockdown of the early COVID-19 pandemic had a beneficial or negative impact on dietary behavior.

Many studies have discussed the relationship between psychological status and eating behaviors during the early COVID-19 pandemic. Some findings suggested that dietary changes were triggered by increased psychological stress during the COVID-19 crisis, and those who report mental stress were at higher risk of increasing their food intake.^[27]^ Anxiety was associated with specific food intake, and increased intake of sugar and saturated fat had been shown to be positively correlated with anxiety.^[16]^ Depressive symptoms during COVID-19 were associated with an increased risk of dietary behavior changes.^[28]^ During the lockdown, not feeling anxious was associated with increased control over overeating.^[24]^ The impact of pandemic-induced stressful events on eating habits should be of concern, as poor eating habits were associated with symptoms of depression and anxiety.^[29]^

Previous articles mainly focused on psychological responses and eating behaviors during the initial COVID-19 pandemic, while few studies focused the phenomenon during the local COVID-19 outbreak in the post-epidemic era. At present, epidemic prevention and control has entered a normal stage in China, but there are still sporadic outbreaks of COVID-19 affecting people life.^[30]^ Therefore, it is of practical significance to explore the psychological status, the dietary changes and their relationship during the local outbreak of COVID-19 in the post-epidemic era. In addition, the results of this paper will provide a reference for Chinese local governments on how to alleviate the psychological pressure and maintain a healthy diet of residents.

The purpose of this paper are to: explore the psychological responses of residents and their influencing factors during the local outbreak of COVID-19 in the post-epidemic era; explore dietary changes and their relationship with psychological responses of residents during the local outbreak of COVID-19 in the post-epidemic era.

## 2. Materials and methods

### 2.1. City selection

Liaocheng, a densely populated city with a population of 6.4 million, is located in the eastern coastal plain of China and in the western part of Shandong Province. Like many other cities in China, Liaocheng has entered the post-epidemic era of normal prevention and control after the initial COVID-19 pandemic controlled. During the period of normal prevention and control, coastal and border cities, densely populated areas, and areas with special locations were prone to local outbreaks of COVID-19, which put considerable pressure on local governments and affected the daily life of residents. Positive cases began to appear in Liaocheng City on August 25, 2022, and then new cases appeared almost every day, showing a local outbreak. Although the number of confirmed cases was less, the source of COVID-19 was not clear, and the strong infectivity of the virus still threatened people life safety and health. In response, the Liaocheng government implemented a series of measures such as: transferring positive patients to designated hospitals for treatment; quarantine and centralized medical observation for close contacts; lockdown for communities or villages with outbreaks; controlling residential quarters, units or companies with close contacts; daily nucleic acid testing for ordinary residents. Residents under quarantine or control were provided daily necessities by volunteers. Three weeks later, the epidemic was basically under control. Communities, villages and companies affected by the local outbreak were gradually unsealed, and residents gradually returned to normal life. Based on the above situation, it is appropriate to choose Liaocheng as a case to explore the psychological reactions and dietary changes of residents during the local outbreak of COVID-19 in the post-epidemic era.

### 2.2. Questionnaire collection

This study used online and anonymous mode to conduct the survey through the software “Questionnaire Star” (Changsha Lanxing Technology, Shanghai, China). Eligible residents were asked to fill in the questionnaire via wechat (Tencent Shenzhen, China) by forming a QR code or a link to the website, which took about 5 to 8 minutes to complete. The questionnaire was first sent to the author relatives, friends, colleagues and classmates, and then the scope of the questionnaire gradually expanded through the form of snowball. A total of 3790 questionnaires were collected and every respondent chose to give informed consent. The investigation took place from September 7 to 14, 2022 (starting from 2 weeks after a few communities or villages were locked down).

### 2.3. Inclusion and exclusion criteria

#### 2.3.1. Inclusion criteria.

Age ≥ 18 years; Living in Liaocheng area for more than half a year; Volunteer to cooperate in the investigation.

#### 2.3.2. Exclusion criteria.

Unable to use mobile phone scanning code to fill in the questionnaire; Answer time<3 minutes.

### 2.4. Content design

The questionnaire was answered in Chinese and contained 5 parts: sociodemographic information, health status, local outbreak related questions, lifestyle changes, anxiety, and depression.

Sociodemographic information included gender, age, occupation (mainly mental work, mainly manual work, else), educational level (primary school or below, junior high school, senior high school/technical secondary school, junior college, and undergraduate or above), marital status, monthly income, monthly food expenditure, place of residence (urban, township, rural).

Health status included self-perceived health status, chronic disease and body mass index (BMI). According to the BMI standard for adults set by the National Health Commission of China, BMI was divided into 4 categories. They were low weight (BMI < 18.5kg/m^2^), normal weight (BMI = 18.5–23.9 kg/m^2^), overweight (BMI = 24.0–27.9 kg/m^2^) and obese (BMI ≥ 28.0 kg/m^2^).

Questions related to the local outbreak of the epidemic included those who experienced lockdown or quarantine during this period, with answer “yes” or “no.” In addition, fear of COVID-19 was also included using the fear of COVID-19 scale, which was composed of 7 questions, with Likert 5-level scale ranging from 1 to 5 points from strongly disagree to strongly agree. The higher the point was, the more serious the fear of COVID-19 was. This study divided it into normal fear and high fear by 19 points.^[31]^

Lifestyle changes included changes in smoking, drinking, and physical activity and dietary changes. The following questions were used to ask: “How did your smoking/drinking/physical activity/dietary changes during the local outbreak compared to the previous period,” all answered with “reduced/unchanged/increased.” Dietary changes were specific to food categories (18 food categories were designed according to the food frequency questionnaire scale). Residents who had changed their diet were asked to choose the reason for the change.

Generalized anxiety disorder-7 (GAD-7) was used for anxiexy. The scale was composed of 7 questions, divided into 4 levels: Did not, 0 points; A few days, 1 point; More than half of the time, 2 points; Almost every day, 3 points. The sum of the points of the 7 questions was the total points, which was divided by 5,10,14, and 19 points to indicate no, mild, moderate, moderate or severe, and severe anxiety.^[32]^ The Cronbach α coefficient was 0.951 in this study. Patient health questionnaire-9 (PHQ-9) was used for depression. This Questionnaire consists of 9 questions and was scored in the same way as GAD-7. According to the limit of 5,10,15, and 20 points, depression was divided into no, mild, moderate, moderate or severe, and severe groups.^[33]^ In this study, the Cronbach α coefficient was 0.932. The Cronbach α coefficient of both scales have been certified internationally. The binary classification of anxiety and depression symptoms was defined as 10 points or higher.^[32,33]^

### 2.5. Statistical analysis

Categorical variables (such as sociodemographic information, health status, lockdown or quarantine, fear of COVID-19, changes in smoking, and drinking and physical activity) were expressed in percentage terms. Numerical variables (such as GAD-7 score and PHQ-9 score) did not meet normal distribution by Kolmogorov-Smirnov test and were expressed as median (quartile). Dietary changes and their causes were plotted as bar graphs. Binary logistic regression model was used to estimate the effects of health status, lockdown or quarantine, fear of COVID-19, changes in smoking, and drinking and physical activity on anxiety status and depression status. Crude model 1 and adjusted model 2 were fitted. Adjustment model 2 was established by controlling demographic variables, including gender, age, occupation, educational level, marital status, monthly income, monthly dietary expenditure, and residence. Kruskal-Wallis H test was used to analyze the distribution of GAD-7 scores and PHQ-9 scores in changes of food intake. Kendall tau-b correlation coefficient was used to explore the relationship between dietary changes and GAD-7 and PHQ-9 scores.

## 3. Results

### 3.1. Basic characteristics

A total of 3790 questionnaires were collected. After removing invalid questionnaires, the final questionnaire included 3562 people, with an effective rate of 93.98%. The samples were mainly women (66.4%), youth (72.5%), married/cohabiting (79.0%), and urban residents (61.5%). The proportion of mainly mental workers was 43.0%, higher than that of mainly manual workers (29.7%). The education level of the surveyed residents were mainly senior high school/technical secondary school or junior high school, accounting for 36.6% and 28.8% respectively. Most residents (70.5%) had a low monthly income of < 5000 yuan. Fifty point 9 persent of residents spend 1000 to 3000 yuan a month on food expenditure. In terms of health status, 77.6% and 75.5% of the residents respectively answered that they were healthy and had no chronic diseases. The number of normal weight (49.4%) was slightly higher than that of overweight (35.0%) (Table 1).

**Table 1:** Sociodemographic information, health status, local outbreak related problems, lifestyle changes, and psychological reactions of residents during the local outbreak of COVID-19 in the post-epidemic era.

	Variable	Level	N	Proportion (%)
Sociodemographic information	Gender	Male	1198	33.6
		Female	2364	66.4
	Age	Youth (18–44)	2581	72.5
		Middle Age (45–59)	955	26.8
		Aged (60 and above)	26	0.7
	Occupation	Mainly mental work	1532	43.0
		Mainly manual work	1058	29.7
		Else	972	27.3
	Degree of education	Primary school or below	112	3.2
		Junior high school	1027	28.8
		Senior high school/ technical secondary school	1305	36.6
		Junior college	420	11.8
		Undergraduate or above	698	19.6
	Marital status	Unmarried	660	18.5
		Married/Cohabiting	2813	79.0
		Divorced or separated	66	1.9
		Widowed	23	0.6
	Monthly income	<5000	2510	70.5
		5000–10000	939	26.4
		>10000	113	3.1
	Monthly food expenditure	<1000	1388	38.9
		1000–3000	1812	50.9
		>3000	362	10.2
	Residence	Urban	2191	61.5
		Township	480	13.5
		Rural	891	25.0
Health status	Self-perceived physical condition	Ill-health	158	4.4
		Average	641	18.0
		Health	2763	77.6
	Chronic disease	Yes	871	24.5
		No	2691	75.5
	BMI	Low weight	159	4.5
		Normal weight	1761	49.4
		Over weight	1245	35.0
		Obese	397	11.1
Local outbreak questions	Lockdown or quarantine	Yes	1348	37.8
		No	2214	62.2
	Fear of COVID-19	Normal fear	1958	55.0
		High fear	1604	45.0
Lifestyle changes*	Smoking frequency**	Increase	57	13.8
		Unchange	208	50.5
		Decrease	147	35.7
	Drinking frequency***	Increase	108	9.4
		Unchange	480	41.6
		Decrease	566	49.0
	Physical activity frequency	Increase	703	19.7
		Unchange	1409	39.6
		Decrease	1450	40.7
Psychological Reaction	GAD-7 score		Median	Quartile
			0	0,4
	PHQ-9 score		Median	Quartile
			2	0,7

BMI = body mass index, COVID-19 = the coronavirus disease 2019, GAD-7 = generalized anxiety disorder-7, PHQ-9 = patient health questionnaire-9.

* Another explanation for dietary changes;

** Delete the number of residents (3150) who have never smoked or quit smoking;

*** Delete the number of residents (2408) who have never drunk or quit drinking.

Residents accounting for 37.8% said they experienced lockdown or quarantine during the local outbreak of COVID-19 and 45.0% said they were highly fear of COVID-19. Compared with those before the local outbreak, the residents with unchanged frequency of smoking (50.5%), decreased frequency of drinking (49.0%) and physical activity (40.7%) accounted for the highest proportion. The medians of GAD-7 score and PHQ-9 score were respectively 0 and 2 (Table 1).

### 3.2. The Influencing factors of anxiety and depression

After controlling demographic variables, binary logistic regression analysis showed that self-perceived physical condition, chronic disease, lockdown or quarantine, fear of COVID-19, frequency of smoking, and drinking and physical activity were influencing factors of anxiety and depression. Self-perceived unhealthy ([adjusted OR of 5.70 [95% CI: 3.38–9.63, *P* < .001]; adjusted OR of 5.37 [95% CI: 3.56–8.10, *P* < .001]]) and average ([adjusted OR of 3.84 [95% CI: 2.71–5.43, *P* < .001]; adjusted OR of 3.19 [95% CI: 2.46–4.15, *P* < .001]]), suffering from chronic disease ([adjusted OR of 2.78 [95% CI: 1.98–3.89, *P* < .001]; adjusted OR of 2.40 [95% CI: 1.86–3.1 0, *P* < .001]]),experiencing lockdown or quarantine ([adjusted OR of 1.38 [95% CI: 1.00–1.90, *P* = .048]; adjusted OR of 1.74 [95% CI: 1.38–2.20, *P* < .001]]), high fear of COVID-19 ([adjusted OR of 4.92 [95% CI: 3.40–7.12, *P* < .001]; adjusted OR of 3.74 [95% CI: 2.91–4.81, *P* < .001]]), reduction of physical activity ([adjusted OR of 1.61 [95% CI: 1.04–2.50, *P* = .033]; adjusted OR of 2.42 [95% CI: 1.70–3.43, *P* < .001]]) were independent risk factors for residents suffering from anxiety and depression. The reduction of smoking frequency ([adjusted OR of 0.15 [95% CI: 0.05–0.50, *P* = .002]; adjusted OR of 0.09 [95% CI: 0.03–0.26, *P* < .001]]) and drinking frequency ([adjusted OR of 0.40 [95% CI: 0.20–0.81, *P* = .01]; adjusted OR of 0.28 [95% CI: 0.17-0.49, *P* < .001]]) was related to the reduction of residents risk of anxiety and depression (Table 2).

**Table 2:** Binary logistic regression analysis was used to analyze the influencing factors of residents’ anxiety and depression during the local outbreak of COVID-19 in the post-epidemic era.

Variable	Level	Anxiety				Depression			
		Crude OR (95% CI)	P	Adjusted OR (95% CI)	*P*	Crude OR (95% CI)	*P*	Adjusted OR (95% CI)	*P*
Self-perceived physical condition^1^			<.001		<.001		<.001		<.001
	Ill-health	5.16 (3.13–8.51)	<.001	5.70 (3.38–9.63)	<.001	4.57 (3.10–6.72)	<.001	5.37 (3.56–8.10)	<.001
	Average	3.60 (2.57–5.04)	<.001	3.84 (2.71–5.43)	<.001	2.85 (2.21–3.67)	<.001	3.19 (2.46–4.15)	<.001
Chronic disease^2^			<.001		<.001		<.001		<.001
	Yes	2.61 (1.91–3.57)	<.001	2.78 (1.98–3.89)	<.001	1.98 (1.57–2.51)	<.001	2.40 (1.86–3.10)	<.001
BMI^3^			.330		0.611		.201		.342
	Low weight	0.86(0.38–1.97)	.724	1.05 (0.44–2.51)	0.922	0.92 (0.51–1.61)	.738	0.87 (0.47–1.60)	.642
	Normal weight	0.70 (0.43–1.14)	.152	0.80 (0.48–1.32)	0.377	0.73 (0.52–1.02)	.068	0.74(0.51–1.05)	.092
	Overweight	0.93 (0.57–1.51)	.754	0.99 (0.60–1.62)	0.958	0.70 (0.49–1.00)	.052	0.74 (0.52–1.07)	.111
Lockdown or quarantine^4^			<.001		<.001		<.001		<.001
	Yes	1.47 (1.08–2.00)	.014	1.38 (1.00–1.90)	0.048	1.75 (1.40–2.19)	<.001	1.74 (1.38–2.20)	<.001
Fear of COVID-19^5^			<.001		<.001		<.001		<.001
	High fear	4.26 (2.97–6.12)	<.001	4.92 (3.40–7.12)	<.001	3.37 (2.64–4.30)	<.001	3.74 (2.91–4.81)	<.001
Smoking frequency^6^			.001		0.001		<.001		<.001
	Unchange	0.26 (0.11–0.62)	.002	0.21 (0.08–0.55)	0.002	0.32 (0.16–0.64)	0.001	0.29 (0.14–0.62)	.001
	Decrease	0.15 (0.05–0.45)	.001	0.15 (0.05–0.50)	0.002	0.11 (0.04–0.28)	<.001	0.09 (0.03–0.26)	<.001
Drinking frequency^7^			.017		0.026		<.001		<.001
	Unchange	0.43 (0.22–0.85)	.015	0.43 (0.21–0.86)	0.017	0.28 (0.17–0.49)	<.001	0.27 (0.16–0.45)	<.001
	Decrease	0.39 (0.20–0.76)	.006	0.40 (0.20–0.81)	0.010	0.26 (0.16–0.43)	<.001	0.28 (0.17–0.49)	<.001
Physical activity frequency^8^			.001		0.001		<.001		<.001
	Unchange	0.87 (0.54–1.39)	.560	0.84 (0.52–1.36)	0.480	1.24 (0.86–1.78)	.258	1.25 (0.86–1.81)	.247
	Decrease	1.67 (1.09–2.57)	.020	1.61 (1.04–2.50)	0.033	2.39 (1.69–3.36)	<.001	2.42 (1.70–3.43)	<.001

1 Based on health;

2 Based on no;

3 Based on obesity;

4 Based on no;

5 Based on normal fear;

6,7,8 Based on the increase.

BMI = body mass index, COVID-19 = the coronavirus disease 2019.

### 3.3. Dietary changes and their causes

**Figure 1.**
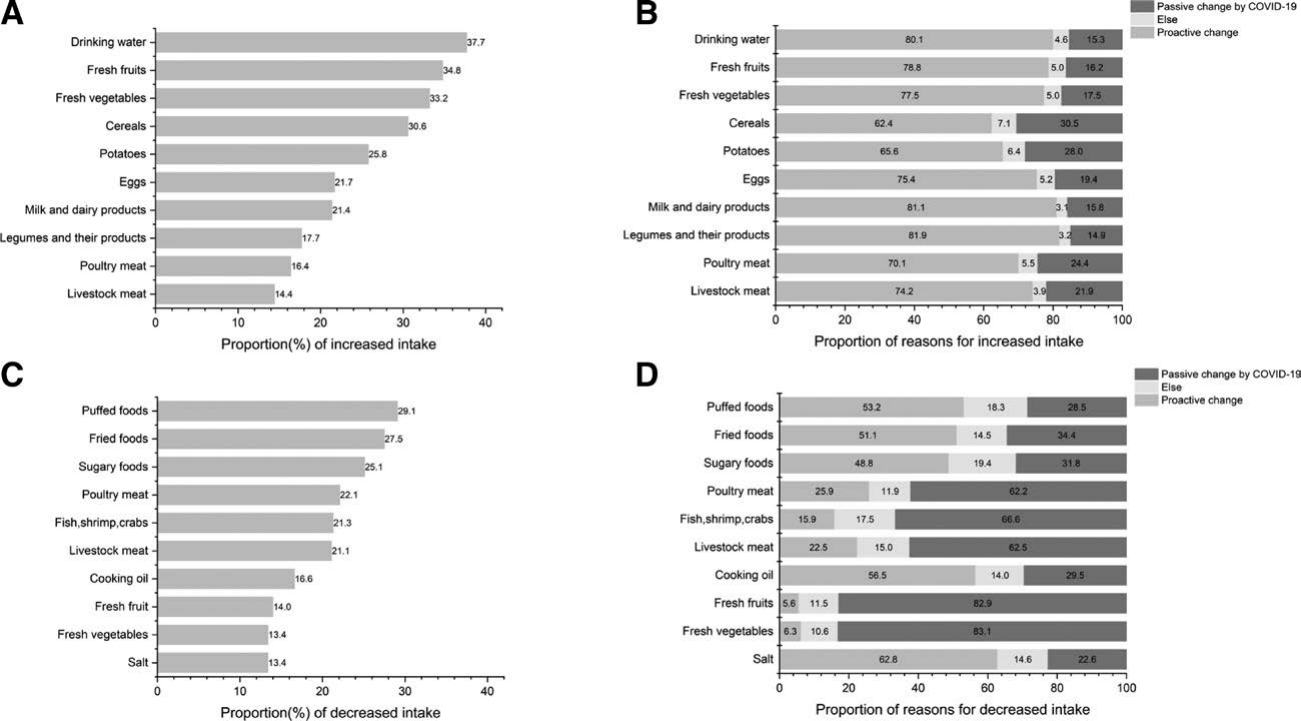
Dietary changes and their causes of residents during the local outbreak of COVID-19 in the post-epidemic era.

Compared with the previous period, the top ten foods intake increased by residents during the local COVID-19 outbreak were drinking water, fresh fruits, fresh vegetables, cereals, potatoes, eggs, milk and dairy products, legumes and their products, poultry meat, and livestock meat, of which drinking water, fresh fruits, and fresh vegetables accounted for the highest proportion, increasing by 37.7%, 34.8%, and 33.2% (Fig. 1A). The main reason for the increase intake of legumes and their products, milk and dairy products, and drinking water was proactive change, accounting for 81.9%, 81.1%, and 80.1%; The main reason for the increase intake of cereals, potatoes and poultry meat was the passive change affected by the local outbreak of COVID-19, with the proportion of 30.5%, 28.0%, and 24.4% (Fig. 1B). The top ten foods intake decreased by residents were puffed foods, fried foods, sugary foods, poultry meat, fish, shrimp and crabs, livestock meat, cooking oil, fresh fruits, fresh vegetables, and salt. The most common types of food were puffed foods, fried foods, and sugary foods, with a reduction rate of 29.1%, 27.5%, and 25.1% (Fig. 1C). Analysing the main reasons, 62.8%, 56.5%, and 53.2% of the residents answered that they were proactive in reducing the intake of salt, cooking oil and puffed foods, while 83.1%, 82.9%, and 66.6% of the residents were forced to reduce the intake of fresh vegetables, fresh fruits, fish, shrimp, and crabs due to the local outbreak in COVID-19 (Fig. 1D).

### 3.4. Relationship between dietary changes and GAD-7 scores and PHQ-9 scores

According to Table 3, except for salt, the intake of other foods (cereals, potatoes, fresh vegetables, fresh fruits, poultry meat, livestock meat, eggs, fish, shrimp and crabs, milk and dairy products, legumes and their products, fungi and algae, nuts, fried foods, sugary foods, puffed foods, and cooking oil and drinking water) had statistically significant distribution in the scores of GAD-7 and PHQ-9 (*P* < .05).

**Table 3:** Relationship between dietary changes and GAD-7 scores and PHQ-9 scores of residents during local COVID-19 outbreaks in the post-pandemic era.

Food species	GAD-7 Test scores for food intake								PHQ-9 Test scores for food intake							
Increase		Unchange		Decrease				Increase		Unchange		Decrease			
P50	P25–P75	P50	P25–P75	P50	P25–P75	*P*	Kendall tau-b	P50	P25–P75	P50	P25–P75	P50	P25–P75	*P*	Kendall tau-b
Cereals	0	0–5	0	0–3.25	3	0–7	<.001	0.048**	2	0–7	2	0-7	5	1–9	<.001	0.046**
Potatoes	0	0–4	0	0–4	2	0–7	<.001	0.047**	2	0–7	2	0-7	4.5	1–9	<.001	0.045**
Fresh vegetables	0	0–3	0	0–4	3	0–7	<.001	0.136**	1	0–5	2	0-7	5	2–9	<.001	0.150**
Fresh fruits	0	0–3	0	0–4	2	0–7	<.001	0.107**	1	0–6	2	0-7	5	1–9	<.001	0.127**
Poultry meat	0	0–5	0	0–3	1	0–6	<.001	0.073**	2	0–7	2	0-7	4	0–8	<.001	0.062**
Livestock meat	0	0–5	0	0–3	1	0–6	<.001	0.070**	2	0–7	2	0-6.25	3.5	0–8	<.001	0.065**
Eggs	0	0–4	0	0–4	2	0–7	<.001	0.063**	2	0–7	2	0-7	5	0–9	<.001	0.060**
Fish, shrimp, crabs	0	0–4	0	0–4	1	0–6	<.001	0.084**	1	0–7	2	0-7	4	0–8	<.001	0.095**
Milk and dairy products	0	0–4	0	0–3	3	0–7	<.001	0.091**	2	0–7	2	0-6	5	0–9	<.001	0.089**
Legumes and their products	0	0–4	0	0–4	2.5	0–7	<.001	0.082**	1	0–7	2	0-7	5	0–9	<.001	0.090**
Fungal and algal foods	0	0–3	0	0–4	2	0–7	<.001	0.102**	0	0–6	2	0-7	4	0–9	<.001	0.109**
Nuts	0	0–4	0	0–4	2	0–7	<.001	0.122**	0	0–6	2	0-7	5	0–9	<.001	0.123**
Fried foods	1	0–6	0	0–4	0	0–4	<.001	-0.026	4	0–8	2	0-7	2	0–6	<.001	-0.042**
Sugary foods	2	0–6	0	0–4	0	0–4	<.001	-0.029*	4	0–8	2	0-7	2	0–7	<.001	-0.034*
Puffed foods	2	0–6	0	0–4	0	0–4	<.001	-0.027	5	0–9	2	0-7	2	0–7	<.001	-0.046**
Cooking oil	1	0–6	0	0–4	0	0–5	0.005	-0.013	4	0–9	2	0-7	2	0–7	0.009	-0.031*
Salt	0	0–6	0	0–4	0	0–5	0.325	0.005	0	0–8	2	0-7	2	0–7	0.575	-0.015
Drinking water	0	0–3	0	0–5	3	0–7	<.001	0.056**	2	0–6	2	0-7	7	1–9	<.001	0.071**

* Significant correlation, *P* < .05;

** Significant correlation, *P* < .01.

COVID-19 = the coronavirus disease 2019, GAD-7 = generalized anxiety disorder-7, PHQ-9 = patient health questionnaire-9.

It was observed that among residents with high GAD-7 scores, the intake of cereals, potatoes, fresh vegetables, fresh fruits, poultry meat, livestock meat, eggs, fish, shrimp and crabs, milk and dairy products, legumes and their products, fungi and algae, nuts and drinking water decreased, and while the intake of sugary foods increased. The decreased intake of cereals, potatoes, fresh vegetables, fresh fruits, poultry meat, livestock meat, eggs, fish, shrimp and crabs, milk and dairy products, legumes and their products, fungi and algae, and nuts and drinking water had a significant positive correlation with the GAD-7 score, but the correlation was weak (*P* < .01, Kendall tau-b = 0.048, 0.047, 0.136, 0.107, 0.073, 0.070, 0.063, 0.084, 0.091, 0.082, 0.102, 0.122 0.056). There was a weak negative correlation between the decreased sugary foods intake and GAD-7 score (*P* < .05, Kendall tau-b = -0.029). Residents had significant differences in the distribution of GAD-7 scores of fried foods, puffed foods and cooking oil, and among residents with high GAD-7 scores, the intake of fried food, puffed food and cooking oil increased. However, no correlation was found between these foods and GAD-7 scores (Table 3).

The residents with high PHQ-9 scores reduced their intake of cereals, potatoes, fresh vegetables, fresh fruits, poultry meat, livestock meat, eggs, fish, shrimp and crabs, milk and dairy products, legumes and their products, fungi and algae, nuts and drinking water, and increased their intake of fried foods, sugary foods, puffed foods and cooking oil. There was a weak significant positive correlation between the PHQ-9 score and the decreased intake of cereals, potatoes, fresh vegetables, fresh fruits, poultry meat, livestock meat, eggs, fish, shrimp and crabs, milk and dairy products, legumes and their products, fungi and algae, and nuts and drinking water (*P* < .01, Kendall tau-b = 0.046, 0.045, 0.150, 0.127, 0.062, 0.065, 0.060, 0.095, 0.089, 0.090, 0.109, 0.123, 0.071). A weak significant negative correlation was observed between decreased intake of fried foods, sugary foods, puffed foods, and cooking oil and PHQ-9 score (*P* < .05, Kendall tau-b = −0.042, −0.034, −0.046, −0.031) (Table 3).

## 4. Discussion

Until now, COVID-19 has lasted for nearly 3 years, and its popularity continues. Although China has entered the stage of normalized prevention and control after the pandemic controlled at the initial stage of the epidemic, local outbreaks occurred from time to time. The impact of various measures taken by the government on residents daily life and mental health cannot be ignored. There were few previous studies on residents psychological reactions and dietary changes in the post-epidemic era. The results of this work revealed the residents psychological status, diet changes and their relationships when the COVID-19 local outbreak occurred in the post-epidemic era. It showed that several factors had an impact on residents anxiety and depression. Residents showed dietary changes in favor of healthier foods. There is a correlation between diet change and anxiety and depression scores.

This study showed that residents of Liaocheng experienced 24.17% mild to severe anxiety and 36.81% mild to severe depression during the local outbreak of COVID-19 in the post-epidemic era, which was similar to previous reports. Li found that during the first COVID-19 outbreak, 27.5% of 3637 participants from 31 provinces in China had mild to severe anxiety and 31.2% had mild to severe depression.^[34]^ In the post- epidemic era, 4.80% of Liaocheng residents had moderate to severe anxiety and 9.63% had moderate to severe depression, which contradicted previous studies. Wang found that 1210 respondents from 194 cities in China, 28.8% reported moderate to severe anxiety symptoms and 16.5% reported moderate to severe depression symptoms at the beginning of COVID-19 outbreak.^[6]^ From this point of view, the overall anxiety and depression symptoms of Liaocheng residents in the post-epidemic era were mostly in the mild range. The level of mild to severe anxiety and depression was roughly consistent with the national level at the beginning of the epidemic, while the level of moderate to severe anxiety and depression was significantly lower than that of the national level at the beginning of the epidemic. The author thought reasons for this phenomenon may be that the rapid and effective prevention and control measures taken by the Chinese government reduced the death rate to a minimum, resumed the production and life in the early stage of the epidemic. In addition, the COVID-19 vaccines were received by most residents during the regular prevention and control period, all of which enhanced their confidence, reduced their pressure, and reduced moderate to severe psychological crisis. However, the repeated outbreaks and local outbreaks still restricted people normal life, the proportion of mild psychological crisis among residents existed relatively high.

After adjusting demographic variables, this work recognized that physical condition, chronic disease, lockdown or quarantine, fear of COVID-19, changes in smoking, and drinking and physical activity were the influencing factors of anxiety and depression in Liaocheng residents during the local COVID-19 outbreak in the post-epidemic era, which were consistent with previous studies. In this study, the more unhealthy you perceived, the more likely you were to suffer from anxiety (average, OR: 3.84; unhealthy, OR: 5.70) and depression (average, OR: 3.19; unhealthy, OR: 5.37), which was consistent with the results of previous studies.^[35,36]^ Suffering from chronic diseases increased the risk of anxiety and depression of residents, which were shown in previous cross-sectional studies, systematic reviews and meta-analysis.^[37–39]^ The risk of anxiety and depression of residents who experienced lockdown or quarantine was 1.38 and 1.74 times higher than that of residents who did not experienced. Wu meta-analysis summarized that during the pandemic period of COVID-19, the risk of anxiety and depression of quarantined people was higher than that of ordinary people,^[38]^ and the research results of Cheng also showed that the prevalence of anxiety or depression in quarantined people was higher during the epidemic period of SARS.^[40]^ The more fear of COVID-19, the higher the risk of anxiety and depression. Previous studies in Pakistan, Israel and Iran have shown that fear of COVID-19 is significantly related to anxiety and depression.^[41–43]^ In this study, based on the increase of smoking and drinking frequency of residents, unchanged and reduced frequency of smoking and drinking were protective factors of anxiety and depression respectively. Previous studies have shown that smoking cessation was related to the improvement of depression and anxiety, while smoking and drinking had a negative impact on anxiety and depression.^[44–48]^ Physical activity reduction was a risk factor for anxiety and depression of residents, which was consistent with previous researches.^[49–51]^ In addition, this work also found that BMI was not significantly related to anxiety and depression, which was inconsistent with the research results of Flanagan EW and Klaser K, who found that BMI was significantly related to anxiety and depression during the epidemic,^[52,53]^ but consistent with the research results of Alves JM and Auny FM. Auny FM found that changes in BMI status were related to depression and anxiety.^[54,55]^

In this work, the residents of Liaocheng City showed a tendency to healthier food when the local COVID-19 outbreak occurred in the post-epidemic era, because the top 3 foods of increased intake were drinking water, fresh fruits and fresh vegetables, and the top 3 foods of decreased intake were puffed foods, fried foods and sugary foods. This shows a good trend, indicating that residents in Liaocheng City had a healthier change in eating behavior during the local outbreak in COVID-19. In China at the beginning of COVID-19, a survey on knowledge, belief and practice of 27 provinces conducted by Luo also showed that residents had a healthier dietary change, with increased intake of vegetables, fruits and water and decreased intake of sugary drinks and snacks.^[19]^ This study was consistent with the results of Luo. Some studies in Iran, Türkiye and Spain also reported that residents turned to healthier food choices during the initial lockdown of COVID-19 epidemic.^[20–22]^ A review of European countries during the COVID-19 pandemic reported that more respondents in all countries announced an increase in the consumption of cereal products, eggs, vegetables and fruits, and a decrease in the consumption of fish and meat, while the consumption of milk, dairy products and legumes in different countries was different.^[56]^ In this study, the consumption of cereals, eggs, vegetables, fruits, fish and meat was consistent with it, while more people reported an increase in the consumption of milk, dairy products and legumes. The intake of Western diet (a diet with high content of saturated fat, sugar and refined carbohydrate) activated the innate immune system, impaired adaptive immunity, stimulated inflammatory response, reduced the host defense against viruses, and increased the susceptibility to COVID-19.^[57]^ During the period of China lockdown at the beginning of the epidemic, the consumption of fruits and vegetables among young people decreased, and the consumption of staple foods other high calorie foods increased.^[58]^ Many citizens of countries reported that they would turn to “comfort food” (food rich in carbohydrates and saturated fat) as a way to relieve anxiety, boredom and other emotions during the lockdown period. If things continued this way, it would not only increase the risk of chronic diseases for citizens, but also increase the medical and economic burden of the country.^[59–61]^ Although this study did not show the tendency to unhealthy food of residents, the local government should still advise people maintaining healthy eating habits to enhance immunity, and reduce the risk of COVID-19 infection and chronic diseases in the epidemic prevention work in the post-epidemic era.^[62]^ When analyzing the reasons for changes in dietary intake, it can be seen that Liaocheng residents were actively changing their intake for more healthy food (such as legumes and their products, milk and dairy products, drinking water, fresh vegetables, and fresh fruits) and less unhealthy food (such as puffed foods, fried foods, sugary foods), while the intake of fresh fruits, fresh vegetables, fish, shrimp and crabs, poultry and livestock meat decreased by a larger proportion due to the impact of the epidemic. For the passively changed food, it may be related to the insufficient supply during the local outbreak,^[58]^ the spread of COVID-19 from cold chain food, and the short shelf life of fresh foods to drive people to buy food with long shelf life.^[61]^

The study observed that there was a correlation between dietary changes and scores of anxiety and depression. The decreased intake of healthy foods (such as cereals, potatoes, fresh vegetables, fresh fruits, poultry meat, livestock meat, eggs, fish, shrimp and crabs, milk and dairy products, legumes and their products, fungi and algae, and nuts and drinking water) was positively correlated with the GAD-7 score and PHQ-9 score. The decreased intake of sugary foods was negatively correlated with the GAD-7 score, and fried foods, sugary foods, puffed foods and cooking oil had a negative correlation with the PHQ-9 score. The interrelationship between diet and mental health has been confirmed many times.^[27,60,63]^ A study in Chile during COVID-19 showed that higher levels of anxiety were related to more consumption of fried foods, cakes, sugary drinks and other foods.^[64]^ In Italy, almost half of the respondents felt anxious, ate comfortable/delicious foods, and tended to increase food intake to feel better.^[65]^ The Israeli survey found that the deterioration of diet quality (increased intake of sugar and saturated fat) during the pandemic was positively correlated with anxiety.^[16]^ Luong TC showed that healthy eating behavior during COVID-19 was a protective factor for anxiety and depression among pregnant women.^[66]^ Therefore, we concluded that healthy diet during the local outbreak of COVID-19 in the post epidemic era was conducive to reducing the level of anxiety and depression of residents, and unhealthy diet would increase the risk of anxiety and depression of residents.

This study has the following advantages: Firstly, this was an Internet based questionnaire survey in the context of public health emergencies and the local outbreak of COVID-19 in the post-epidemic era. It could obtain enough samples in a short time and avoid crowd contact. So it was a safe research form. Secondly, the study was carried out quickly 2 weeks after the lockdown of some communities or villages. The GAD-7 and PHQ-9 scales were selected to collect the emotional problems within 2 weeks, which were investigated in the critical period of local outbreak in the post epidemic era.

This study has the following shortcomings: Firstly, this was a cross-sectional survey, which only provided clues for the government to formulate public health strategies, promote healthy diet of residents, and maintain mental health. It could not draw a conclusion of causality. Cohort studies will be needed in the future. Secondly, the common difficulty of online nutrition questionnaire survey was that it was difficult to reliably investigate the food intake. The nutrition part of this questionnaire survey did not design quantitative indicators, only contained qualitative indicators of intake changes, so it could not compare the specific values of changes before and after. Thirdly, for the convenience of sampling, the snowball method used in the online questionnaire led to selection bias. The respondents were subjective when answering questions, and would inevitably be affected by recall bias. In addition, the height, weight and other values were self-reported results, and no objective measurement had been carried out, which may cause actual misstatement, leading to people concern about reliability. Finally, the results of the investigation could not but be cautiously generalized to the overall population in China, as the scope of the survey was limited to areas with localized outbreaks.

## 5. Conclusion

Our results provide a basis for the local government to better formulate epidemic prevention policies and carry out public health practices in the post epidemic era. While implementing local lockdown or quarantine to ensure the safety of residents lives, the local government should also pay attention to the psychological reaction and healthy diet of residents during this period. We found that self recognition of physical condition, chronic disease, lockdown or quarantine, fear of COVID-19, changes in smoking, drinking and physical activity had an impact on residents psychology. The local government should optimize lockdown measures, correctly guide residents views on COVID-19, advocate smoking and drinking cessation, and promote moderate physical activity. Individual residents should relax, exercise reasonably every day, improve their physical fitness and reduce the occurrence of chronic diseases. In our results, residents preferred healthy diet. The Chinese government has formulated the Outline of the “Healthy China 2030” Plan to improve peoples health. Our results show a good trend. Local governments should continue to strengthen and consolidate, publicize the importance of healthy diet, and ensure the nutritional status of residents. When analyzing the reasons for the changes in diet, we found that the main reason for the decreased intake of food that difficult to preserve, such as fresh fruits, fresh vegetables, fish, shrimp and crabs, poultry meat, and livestock meat, was due to the local outbreak of the epidemic. In view of this, the local government should target the appropriate supply of such food to quarantined residents, instead of just supplying staple food to maintain energy. They should also take measures to ensure the smooth cold chain transportation link and not affect the purchasing choices of ordinary residents. Individual residents should change their concept of buying food with a long shelf life and believe that the local government can control the outbreak of the epidemic in a short time. Finally, our results show that healthy diet is negatively related to anxiety and depression, while unhealthy diet is positively related to anxiety and depression. Therefore, it is necessary for the government to formulate public health policies and strengthen publicity to raise residents awareness of healthy diet and mental health, to help residents eat properly and reduce their psychological burden in the post-epidemic era.

## Author contributions

**Conceptualization:** Liyong Chen.

**Data curation:** Luying Qi.

**Formal analysis:** Luying Qi, Qingtao Yu.

**Funding acquisition:** Zhengyan Liang, Liyong Chen.

**Investigation:** Luying Qi, Qingtao Yu, Zhengyan Liang, Yang Lu.

**Methodology:** Luying Qi.

**Project administration:** Liyong Chen.

**Resources:** Luying Qi.

**Software:** Liyong Chen.

**Supervision:** Liyong Chen.

**Validation:** Qingtao Yu, Liyong Chen.

**Visualization:** Luying Qi, Zhengyan Liang.

**Writing – original draft:** Luying Qi, Qingtao Yu, Zhengyan Liang, Yang Lu, Zhihua Ma, Chujie Hou, Zhiyong Zhu.

**Writing – review & editing:** Zhihua Ma, Chujie Hou, Zhiyong Zhu, Liyong Chen.
